# Worldwide trends in scientific publications on association of gut microbiota with obesity

**DOI:** 10.22038/ijbms.2018.30203.7281

**Published:** 2019-01

**Authors:** Hanieh-Sadat Ejtahed, Ozra Tabatabaei-Malazy, Ahmad-Reza Soroush, Shirin Hasani-Ranjbar, Seyed-Davar Siadat, Jeroen Raes, Bagher Larijani

**Affiliations:** 1 Obesity and Eating Habits Research Center, Endocrinology and Metabolism Clinical Sciences Institute, Tehran University of Medical Sciences, Tehran, Iran; 2 Non-Communicable Diseases Research Center, Endocrinology and Metabolism Population Sciences Institute, Tehran University of Medical Sciences, Tehran, Iran; 3 Endocrinology and Metabolism Research Center, Endocrinology and Metabolism Clinical Sciences Institute, Tehran University of Medical Sciences, Tehran, Iran; 4 Department of Mycobacteriology and Pulmonary Research, Microbiology Research Center, Pasteur Institute of Iran, Tehran, Iran; 5 Department of Microbiology and Immunology, Rega Institute, KU Leuven, Leuven, Belgium

**Keywords:** Bibliometrics, Endotoxemia, Gut flora, Gut microbiota, Obesity

## Abstract

**Objective(s):**

Recent evidence has shown underlying roles of gut dysbiosis and metabolic endotoxemia in obesity and its complications. Despite the large number of experimental and clinical researches performed on gut microbiota and obesity, no bibliometrics’ study has been conducted so far. We aimed to assess the trend of global scientific publications in the field of gut microbiota and obesity.

**Materials and Methods:**

The bibliometrics’ data from January 2000 to April 2017 were retrieved based on Scopus database. The analysis of the publication year, main source, citation, subject area, co-authorship network, and geographical distribution were carried out, accordingly. The data were analyzed using the Scopus analysis tools, SPSS version 15 and Visualizing Scientific Landscapes (VOS) viewer version 1.6.5.

**Results:**

Out of 4384 documents that were identified, the United States published the highest number (28.2%), followed by China and United Kingdom. The number of publications showed an increasing trend over the years of which the most productive year was 2016. The leading subject area was medicine. Most of published scientific documents were original articles and the top source was “PLoS One”. The documents were cited totally 153576 times with average citations per article as 35.03, and h-index of 159. Top author in the co-authorship network assessment was “Wang J.” from China.

**Conclusion:**

This study could provide practical sources to researchers to find highly cited studies. Moreover, the study could pave the way for researchers to be engaged in studies which potentially lead to more publication in the field.

## Introduction

The prevalence of obesity, as one of the most important health problems influenced by environmental and genetic factors, has been increasing worldwide, both in developed and developing countries (1-3). Although obesity increases the risk of developing serious health conditions, there is no successful method for its control up to now (4). Multiple socio-economic, behavioral, and biological determinants have been known as influencing factors in the prevention and management of obesity. Current researches have focused on the basic aspects of obesity and the emerging science for its control (5). Microbiota, as one of the hot topics in the research field of obesity pathology and management has strong supports either from national institutes or pharmaceutical companies. Gut microbiota that is the microbial community inhabiting the intestine plays as an endocrine organ and its imbalance is related to numerous disorders (6). The gut microbiota might affect energy balance through several mechanisms including the fermentation of indigestible dietary compounds, secretion of complex biochemical compositions, impacting on intestinal permeability and interference with metabolic pathways (6-8).

The rationale for ongoing efforts in investigating the relationship of gut microbiota and obesity is the development of monitoring tools and the discovery of new treatment and preventive strategies (9, 10). Despite the large number of experimental and clinical researches performed on gut microbiota and obesity, no bibliometrics’ study has been yet conducted. The bibliometric analysis, which is an effective method to evaluate the current research performance, could provide practical information for basic researchers, care givers and health authorities engaged in microbiota research to conduct better studies and accelerate therapeutic innovations (11, 12). 

The aim of the present study is to assess the trend of global scientific publications in field of gut microbiota and obesity with a focus on aforementioned points. 

## Materials and Methods

***Data source***

A descriptive bibliometric study of scholarly products covering the role of gut microbiota in obesity was conducted in Scopus database available at http://www.scopus.com. An all-embracing coverage of the Scopus database in health and biomedical fields as well as its high coverage of citation reports and its easy access to various valid analytical tools made it a suitable choice for our study (13). 

***Search strategies***

Articles were searched in Scopus database across title, abstract and keywords using the following queries: (gut  AND  (microbiot*  OR  bacteria  OR  microbiome )  OR  prebiotic  OR  probiotic  OR  (intestin*  AND  (flora  OR  bacteria  OR  microbiot*  OR  microbiome))  OR  antibiotic  OR  dysbiosis)  AND  (( obes*  OR  adipos*  OR  anthropometric  OR  overweight  OR  weight  OR  (body  AND  (size  OR  composition  OR  mass))  OR  BMI  OR  (fat  AND  mass)). The above key words were chosen from the list of Medical Subject Headings (MeSH) provided by the National Library of Medicine (NLM) /PubMed.

**Figure 1:**
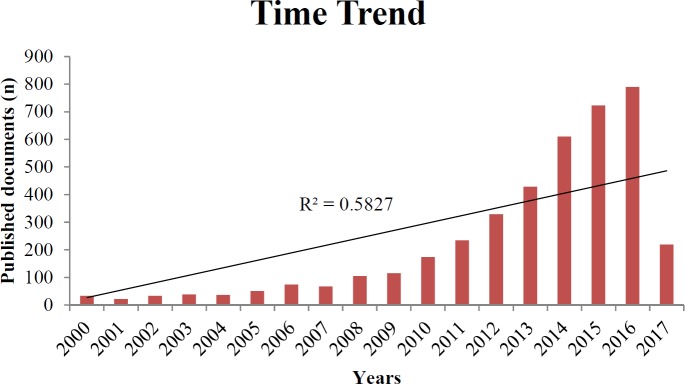
Time-trend distribution of published documents in field of gut microbiota and obesity

**Figure 2:**
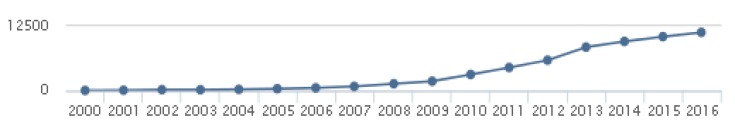
Chart of citation of published documents in our study

**Figure 3:**
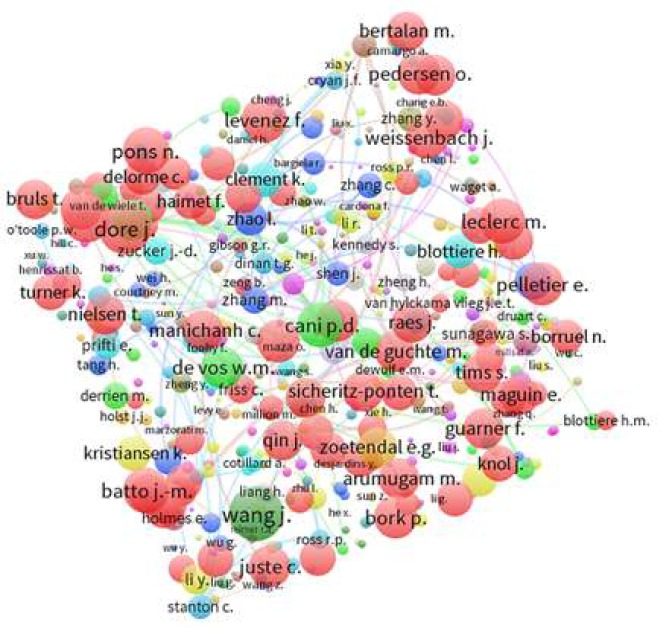
Map (label view) of co-authorship network of the authors published scientific documents in field of gut microbiota and obesity

**Figure 4:**
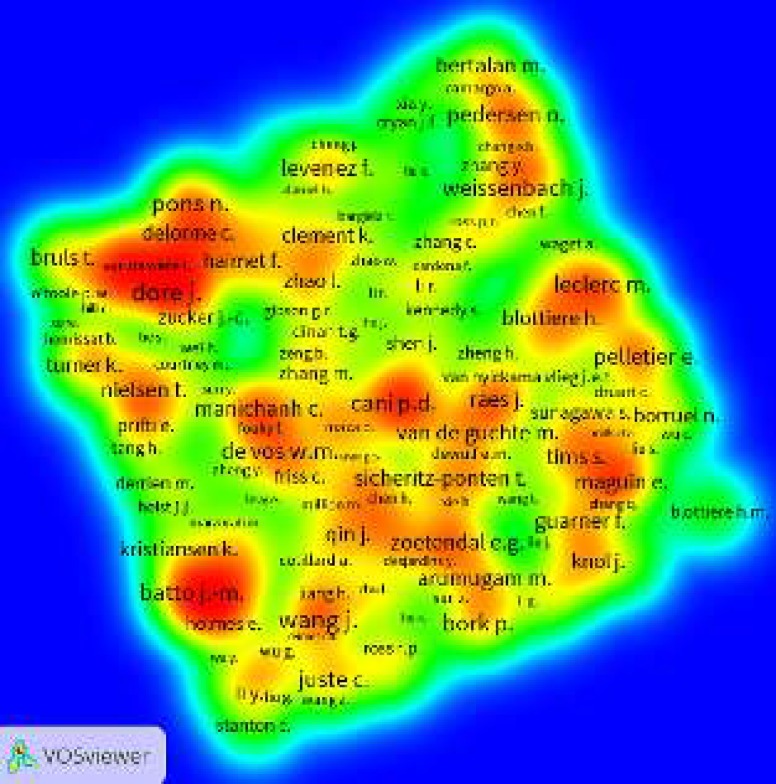
Density view of co-authorship network of the authors published scientific papers in field of gut microbiota and obesity

All relevant publications in the field of gut microbiota and obesity published before April 2017 were included in the analysis with no language limitation. Studies in the subject areas of veterinary, poultry science, soil biology, dentistry, engineering, material science, humanity and art science, computer and mathematics were excluded. 

***Data analysis***

The collected data were publication year, main source (journal) with its impact factor, author’s name and affiliation, geographical distribution, document’s type and language, subject area, and document’s citations which were retrieved and analyzed using the Scopus database. The impact factors (IF) of the journals that were retrieved from the Journal Citation Report (JCR) available at https://jcr.incites.thomsonreuters.com was used to compare the relative importance of journals within a specific field (14, 15). Moreover, the h-index was utilized to estimate the quality and impact of research documents. H-index could be determined based on the number of citations that each document received in other publications (16).

**Table 1:** Characteristics of top 10 sources for the published documents in field of gut microbiota and obesity.

**Title of journal**	**IF (SJR)**	**Documents (number/percent)**	**Total citations to document**	**Citation per document**	**Citation to highest** **cited document**
Plos One	3.057 (1.395)	186 (4.24)	5399	28.87	624
British Journal of Nutrition	3.311 (1.587)	95 (2.17)	3879	40.83	634
Gut Microbes	(1.473)	60 (1.37)	849	14.15	135
Scientific Reports	5.228 (2.073)	53 (1.21)	283	5.34	42
Journal of Nutrition	3.740 (2.040)	52 (1.19)	4327	83.21	1964
World Journal of Gastroenterology	2.787 (1.076)	45 (1.03)	872	19.38	101
Applied and Environmental Microbiology	(1.891)	42 (0.96)	2688	64	431
Proceedings of the National Academy of Sciences of the United States of America	9.423 (6.883)	38 (0.87)	9899	260.5	2007
Nature	38.138 (21.936)	36 (0.82)	21701	602.81	3332
Nutrients	3.759 (2.275)	36 (0.82)	749	20.81	219

**Legend: IF**: Impact factor, **SJR:** SCImago journal rank.

**Table 2:** Characteristics of top 10 institutes’ affiliation for published documents in field of gut microbiota and obesity

**Rank**	**Name of institute**	**Documents ** **(number, percent)**	**Country**
1	Universite Catholique de Louvain	101 (2.3%)	Belgium
2	Kobenhavns Universitet	83 (1.89%)	Denmark
3	Inserm	64 (1.46%)	France
4	Goteborgs Universitet	64 (1.46%)	Sweden
5	University College Cork	62 (1.41%)	Ireland
6	National University of Ireland, Cork, Alimentary Pharmabiotic Centre	59 (1.34%)	Ireland
7	University of Reading	57 (1.3%)	United Kingdom
8	Imperial College London	57 (1.3%)	United Kingdom
9	Wageningen University and Research Centre	54 (1.2%)	Netherlands
10	VA Medical Center	51 (1.16%)	USA

**Table 3:** Characteristics of top 10 highly cited published documents in field of gut microbiota and obesity

**Rank**	**Name of article**	**Number of citations**	**Year **	**Type of article**	**Country**		**Title of journal**	**IF (SJR)**
					**Corresponding author**	**Co- Authors**		
1	An obesity-associated gut microbiome with increased capacity for energy harvest	3312	2006	Original article	USA	USA	Nature	38.138
2	A human gut microbial gene catalogue established by metagenomic sequencing	3098	2010	Original article	China, France, Denmark	International	Nature	38.138
3	A core gut microbiome in obese and lean twins	2596	2009	Original article	USA	USA	Nature	38.138
4	Microbial ecology: Human gut microbes associated with obesity	2555	2006	Original article	USA	USA	Nature	38.138
5	The gut microbiota as an environmental factor that regulates fat storage	1993	2004	Original article	USA	International	Proceedings of the National Academy of Sciences of the United States of America	9.423
6	Obesity alters gut microbial ecology	1925	2005	Original article	USA	USA	Proceedings of the National Academy of Sciences of the United States of America	9.423
7	Host-bacterial mutualism in the human intestine	1916	2005	Review	USA	USA	Science	34.661
8	Metabolic endotoxemia initiates obesity and insulin resistance	1655	2007	Original article	France	International	Diabetes	8.095
9	Enterotypes of the human gut microbiome	1373	2011	Original article	Germany, France	International	Nature	38.138
10	Changes in gut microbiota control metabolic endotoxemia-induced inflammation in high-fat diet-induced obesity and diabetes in mice	1242	2008	Original article	France	International	Diabetes	8.095

**IF**: Impact factor, **SJR:** SCImago journal rank.

Analysis of the extracted data was performed through ‘Analyze search results’ function of Scopus web databases. SPSS software, version 15 (SPSS Inc., Chicago, IL, US) was used to assess the correlation between the number of published papers and the year of publication. By usage VOS viewer (Visualizing Scientific Landscape) software, version 1.6.5 was assessed scientific collaboration between authors with published papers in the field (17). The co-authorship mapping and clustering information could be provided by VOS viewer, available at www.vosviewer.com. 

## Results

***Time trend in publications***

Overall, 4384 documents, published from January 2000 to April 2017 were analyzed. The time-trend distribution of publications is shown in Figure 1. Publications in the field of the gut microbiota and obesity have increased significantly over the recent years. The highest number of documents has published in 2016 (790 documents, 18%). The overall association between the number of published documents and the year of publication is 0.806 with *P-*value <0.001. The R-squared value of 0.583 suggests a steady and significant increase over the same period. Details are shown in Figure 1.

***Subject area, type, language, and main source of documents***

Among the subject area of the documents, top five are medicine (66.3%), biochemistry, genetics and molecular biology (31.7%), agricultural and biological sciences (22.6%), immunology and microbiology (17.4%), and nursing (12.7%).

Most of our analyzed documents are original articles (3039 documents, 69.3%) that followed by 969 review articles (22.1%), and 102 conference papers (2.3%). The remaining papers are letters, editorials, short surveys and notes (184 documents, 4.2%). Top ten languages of scientific publications are English, French, Chinese, German, Spanish, Japanese, Russian, Polish, Czech and Portuguese, respectively which most of them published in English language (96.1%). 

Table 1 shows the characteristics of the main journals (sources). The “Plos One” with 186 documents has been scored as the first rank followed by “British Journal of Nutrition”, “Gut Microbes”, “Scientific Reports”, “Journal of Nutrition”, “World Journal of Gastroenterology”, “Applied and Environmental Microbiology”, “Proceedings of the National Academy of Sciences of the United States of America”, “Nature” and “Nutrients”, respectively. The impact factor of all top 10 journals, except for “Gut Microbes” and “Applied and Environmental Microbiology” is remarkable (18).

***Geographical distribution***

Considering the geographical distribution of published articles, the United States is the most productive country with 1237 documents (28.2%). China is ranked as the second (9%) and the United Kingdom, France, Germany, Italy, Canada, Spain, Sweden and Belgium are ranked subsequently as the top 10 countries with a high number of published documents (3554 documents/ 81.07% of total published papers). 

***Authors and Institutes’ characteristics of published documents***

We found 159 authors as the first-author with published articles in the field of gut microbiota and obesity. Top 10 authors who have the most number of publications in this field are Cani PD with 86 documents followed by Delzenne NM (65 documents), Backhed F. (51 documents), Gordon JI and Shanahan F (each one with 34 documents), Neyrinck AM, Gibson GR, Raoult D, Everard A, and Cryan JF with 33, 31, 30, 28, and 26 documents, respectively. Out of above top 10 authors, four authors are from Belgium, two authors from Ireland, and others are from Sweden, USA, United Kingdom, and Canada.

The first ranked institute for publications in the field of gut microbiota and obesity is “Universite Catholique de Louvain”, followed by “Kobenhavns Universitet” having the second rank and “Inserm” and “Goteborgs Universitet”, both owning the third rank. The details of findings are shown in Table 2. 

***Citation number***

The trend of citation number is shown in Figure 2. Total citation number of 4384 documents is 153576 times at the time of data analysis (until the fourth April 2017). Therefore, the average number of citations per article is 35.03. Citations in the field of the gut microbiota and obesity have increased over the time with the highest number of citations being 33911 times in 2016. 16.95% of all documents (743 papers) don’t receive any citation until the date of analysis.

The h-index for analyzed documents is 159 indicating that 159 documents have cited at least 159 times. Characteristics of top 10 highly cited published documents in the field of gut microbiota and obesity are summarized in Table 3. 

The citation number of these top 10 articles ranged from 3312 to 1242 times. The highest cited paper is an original article entitled “An obesity-associated gut microbiome with increased capacity for energy harvest”, been cited 3312 times. The second ranked article entitled “A human gut microbial gene catalogue established by metagenomic sequencing” has 3098 citations. The third rank article is “A core gut microbiome in obese and lean twins” with 2596 citation numbers at the time of our study. 

As shown in Table 3, of top 10 highly cited documents, 9 papers are original articles and 1 paper is a review, entitled “Host-bacterial mutualism in the human intestine”. Of these top 10 articles, 5 documents are published in Nature, 2 documents in each of Diabetes and Proceedings of the National Academy of Sciences of the United States of America and 1 document in Science. The Washington University School of Medicine (USA) with 5 articles from 10 highly cited documents is ranked as the first. 

***Co-authorship network mapping***

In this part of the study, VOS viewer software is used to assess the co-authorship network of authors. In order to map this network, the minimum number of documents published by an author is considered as 2 papers. Out of 16351 authors, 3561 authors meet this threshold. After excluding 101 authors with no co-authorships, the remaining authors are analyzed. The labels and density views of co-authorship network of authors in field of gut microbiota and obesity are shown in Figure 3 and Figure 4, respectively. Top 10 authors in the field based on co-authorship networks of authors are “Wang J” (444 co-authorships) followed by “Dore j”, “Ehrlich S”, “Pons N”, “Cani PD”, “Levenez F”, “Batto JM”, “Leclerc M”, “Bork P” and “Pedersen O”, each one with 411, 348, 339, 329, 327, 323, 323, 297, and 297 co-authorships, respectively (Figure 3). According to Figure 4, the highest density in the network is attributed to “Wang J” from China.

## Discussion

In the present study, the trend of global scientific publications in the field of the gut microbiota and obesity was assessed. In this view, 4384 documents were extracted from the Scopus database from January 2000 till April 2017. Although, the documents that were indexed outside of the Scopus database were not included in this analysis, it should be mentioned that Scopus search engine covers an acceptable number of publishers and peer-reviewed journals in different fields (19). In addition, in a study that compared the citations’ reports in three essential web databases including Web of Science, Scopus, and Google Scholar, a high coverage of citation reports was shown for Scopus (20). Thus, this study could give a reliable view about the characteristics of research in the field of gut microbiota and obesity. 

The number of publications in this field showed an increasing trend over the years marking 2016 as the most productive year. This progressive increase indicated an augmented interest in this topic to promote the development of novel therapeutic strategies like the application of probiotics and prebiotics for human health. It could be assumed that major financial investments including Human Microbiome Project (HMP) and Metagenomics of the Human Intestinal Tract (MetaHIT) had an impact on research output in the field of gut microbiota and obesity in the United States and Europe. The HMP was established by the United States National Institutes of Health (NIH) as a conceptual extension of the Human Genome Project in 2007 with the aim of identifying and characterizing the human microbiota in health and disease states (21-23). MetaHIT was known as a European Union Project that launched by the European Commission to explore the associations between microbial genes and human phenotypes, especially targeted obesity and inflammatory bowel diseases. This project was implemented in order to compare the gut microbiota of healthy and sick individuals, follow up patients in clinical remission, conduct nutritional interventions, and compare gut microbiota of patients responding or not to a drug (24, 25). Furthermore, the advent of next generation sequencing (NGS) technology in microbiota analysis allowed more sophisticated microbiota studies over the previous culture-based and molecular methods and led to significant growth in the number of literature in the field. To put it in another way, one reason for the upward trend in the number of articles could be attributed to the emergence of this technology and the increasing interest of scientists in this field (25-28). 

Generally, a relatively small number of countries were responsible for the majority of research on gut microbiota and obesity. The United States as the leading country published the most articles and China ranked the second. It should be noted that although the United States was published nearly one third of articles in this field, “Universite Catholique de Louvain” from Belgium was found as the top institute focused on this topic. This could be well explained considering the fact that several research entities in the USA are working in this field; however, the majority of research in this area in Belgium is conducted in Universite Catholique de Louvain and Belgium have good collaboration with other countries. 

The majority of published documents in this field were original articles (69.3%). This observation could be related to challenge of the global epidemic of obesity for the public health services and increasing efforts to combat this problem. The top subject area of published documents was medicine that followed by biochemistry, genetics and molecular biology studies refer to the interests not only for better understanding the signaling pathways on the association of gut microbiota with obesity, but also on the role of gut microbiota, as a new target therapy for obesity. Dietary manipulation of the gut microbiota such as prebiotics and probiotics could affect the gut microbiota composition. However, responses of obese subjects to these manipulations are varied. Analysis of the microbiome in obese individuals could be added to future routine personalized medicine, which leads to the development of novel therapeutic strategies (29).

The majority of articles were published in high impact and high quality international journals indicating the worldwide attention to this topic. Among the top 10 sources, “Nature” with an IF of 38.138 was shown to present the importance of new findings about gut microbiota. The “PLoS One” with the highest number of papers in this field was indexed in many of the important citation databases such as PubMed, MEDLINE, Web of Science, and Scopus. 

Citations in the field of gut microbiota and obesity had increased over the time with the highest number of citations in 2016, indicating the progressive appealing of the medical professionals and researchers to this topic. The h-index of 159 for analyzed documents was considered as the good quality of the articles confirming the accepted visibility of the article. The 10 top-cited articles were published from 2004 to 2011. The United States with 5 articles from 10 highly cited documents was ranked as the first. It should be noted that the other half of these studies were multinational collaboration. It could show the multidisciplinary efforts in this field. The most cited article (3312 times) was published by Turnbaugh *et al* from the Washington University in 2006, entitled “An obesity-associated gut microbiome with increased capacity for energy harvest” (30). This study published in “Nature” with an IF of 38.138 and SJR of 21.936. 

It was shown that co-authorship network is the best bibliometric indicator to demonstrate different co-authorship patterns (31). Co-authorship networks could show research collaborations between different academic institutions. Top author in the co-authorship network assessment was “Wang J” from China. Analysis of the label and density views of co-authorship network of authors that showed 6 authors of the top 10 authors were French, showing that French researchers had good international collaboration in the field of obesity and gut microbiota. 

Our study had some strengths and limitations. First, we focused on specific subjects on scholarly products in the field of gut microbiota and obesity. Second, we used Scopus web database that has a high multidisciplinary coverage in science. Third, we assessed the worldwide trends of scientific publications concomitant draw international collaboration and co-authorship network of authors and institutions. The potential limitation of this study was related to the database used to retrieve relevant documents. Included database could not represent all published scientific journals; in fact, some highly cited scientific publications may appear in journals other than those indexed in Scopus. In addition, given the fact that the universities consisted of several research centers, unfortunately, it was not possible for us to distinguish these centers in the present study. 

## Conclusion

The results of the present scientometric study showed an ascending trend in global publications on the relationship between gut microbiota and obesity over the years. The trend was in line with the development of novel diagnostic tools and helped identifying approaches to the modulation of the individual’s microbiota to promote health and manage obesity. Evaluation of studies in this field was mentioned necessity to perform analysis of gut microbiota of different countries’ population regarding the effect of geography, race and environmental factors on gut microbiota. This bibliometric analysis could be used to explore research productivity over time, map research needs in this field, and identify funding research centers to produce high-quality documents. It could also highlight the highest contributions of the academic institutions on the subject matter. Overall, this study could provide practical sources for researchers to find highly cited studies. Additionally, the study could pave the way for researchers to be engaged in studies potentially lead to more publications in this field. 
